# Moderating Effects of Harm Avoidance on Resting-State Functional Connectivity of the Anterior Insula

**DOI:** 10.3389/fnhum.2018.00447

**Published:** 2018-11-12

**Authors:** Ashley A. Huggins, Emily L. Belleau, Tara A. Miskovich, Walker S. Pedersen, Christine L. Larson

**Affiliations:** ^1^Department of Psychology, University of Wisconsin-Milwaukee, Milwaukee, WI, United States; ^2^Center for Depression, Anxiety and Stress Research, McLean Hospital/Harvard Medical School, Belmont, MA, United States; ^3^Center for Healthy Minds, University of Wisconsin-Madison, Madison, WI, United States

**Keywords:** functional connectivity, harm avoidance, anxiety, resting-state, personality

## Abstract

As an index of behavioral inhibition and an individual’s propensity to avoid, rather than seek, potentially dangerous situations, harm avoidance has been linked to internalizing psychopathology. Altered connectivity within intrinsic functional neural networks (i.e., default mode [DMN], central executive [CEN] and salience networks [SN]) has been related to internalizing psychopathology; however, less is known about the effects of harm avoidance on functional connectivity within and between these networks. Importantly, harm avoidance may be distinguishable from trait anxiety and have clinical relevance as a risk factor for internalizing psychopathology. A sample of young adults (*n* = 99) completed a resting state functional magnetic resonance imaging (fMRI) scan and self-report measures of harm avoidance and trait anxiety. Whole brain seed-to-voxel and seed-to-network connectivity analyses were conducted using anterior insula seeds to examine associations between harm avoidance/trait anxiety and connectivity. After adjusting for sex and age, there was a significant negative effect of harm avoidance on connectivity between the anterior insula and clusters in the precuneus/posterior cingulate cortex (PCC) left superior/middle frontal gyrus, dorsal anterior cingulate cortex (dACC) and bilateral inferior parietal lobule (IPL)/angular gyrus. Seed-to-network analyses indicated a negative effect of harm avoidance on connectivity between the right anterior insula and anterior and posterior DMN. There were no effects of trait anxiety on functional connectivity of the anterior insula. Overall, the results indicate that individual differences in harm avoidance relate to disruptions in internetwork connectivity that may contribute to deficits in appropriately modulating attentional focus.

## Introduction

Harm avoidance, a personality trait reflecting individuals’ propensities to avoid potentially dangerous situations, has consistently been observed in depressive (Abrams et al., 2004; Smith et al., 2005) and anxious psychopathologies (Starcevic et al., 1996; Ettelt et al., 2008; Wachleski et al., 2008). More specifically, harm avoidance has been characterized as a motivational tendency that subserves approach-avoidance behaviors, independent of psychopathology (Tellegen and Waller, 2008; Sylvers et al., 2011). For instance, harm avoidance is thought to reflect hypersensitivity to danger, while, in contrast, general anxiety may correspond to a vague sense of fear and desire to escape that may not have an identifiable source (Tellegen and Waller, 2008). As such, harm avoidance may be characterized better as trait fear, rather than trait anxiety (Sylvers et al., 2011). Individual differences in harm avoidance have been proposed to be consequent to variability in arousal regulation, which may further indicate susceptibility to affective disorders (Zuckerman and Kuhlman, 2000; Hariri et al., 2005). Thus, given the clinical relevance of this form of behavioral inhibition, examining individual differences in harm avoidance and its supporting neurobiological features may provide insight into mechanisms of risk for internalizing psychopathology.

Although the neural systems implicated in anxiety and depression have been studied extensively (see Etkin and Wager, 2007; Hamilton et al., 2012), less is known about which neural circuits are uniquely linked to psychopathology and which may relate to personality traits, such as harm avoidance, that are relevant to internalizing symptoms. As personality describes individuals’ persistent behavioral response patterns, it is likely that these traits may be reflected in the brain’s functional architecture, and may be distinguishable from the patterns observed in relation to internalizing symptomatology. Moreover, neural signatures of harm avoidance that are separable from actual symptoms may have implications on risk for internalizing psychopathology, playing a mechanistic role. Specifically, as harm avoidance describes a persistent pattern of avoidance in response to dangerous, fear-inducing situations, this behavioral pattern could exacerbate internalizing symptoms as highly harm avoidant individuals fail to learn avoided situations may be safe or rewarding and, subsequently, become more fearful or inhibited. However, to date, insight into such a pattern has remained elusive, as limited research on neurobiological correlates of harm avoidance has also been muddied by differences in construct definition (Tellegen, 1985; Cloninger et al., 1993; Tellegen and Waller, 2008). Broader definitions of harm avoidance have contributed to overlapping constructs that somewhat conflate trait anxiety and trait fear; yet, it has been proposed that these constructs are distinct and supported by different neurobiological systems (Sylvers et al., 2011).

Examination of the brain’s intrinsic functional architecture may help elucidate the neural systems unique to harm avoidance. To this end, neuroimaging research has revealed spatially distinct, anti-correlated networks—the default mode network (DMN) and central executive network (CEN)—that support different cognitive processes (Fox et al., 2005; Damoiseaux et al., 2006). The DMN consists of a number regions demonstrated to be active during wakeful, resting states and includes key nodes such as the medial prefrontal cortex (mPFC), posterior cingulate cortex (PCC), and precuneus (Buckner et al., 2008). These neural regions generally subserve internally focused or cued processes, including self-referential processing, thinking about others and episodic memory (Buckner et al., 2008; Uddin et al., 2008). In contrast, the regions comprising the CEN have been shown to come online during performance of cognitively demanding tasks. This network includes a set of regions consistently engaged during processes requiring endogenous attention and goal-directed task performance, such as the dorsolateral PFC and posterior parietal cortex (PPC; Fox et al., 2006; Seeley et al., 2007; Sridharan et al., 2008). Taken together, the DMN and CEN demonstrate an antagonistic relationship, wherein increases in regions of one network correspond to proportionate decreases in the other (and vice versa) and are dependent on cognitive demands and task difficulty.

Disruption within these networks has been related to various disease states, traits and overt behaviors (Adelstein et al., 2011); insular dysfunction within the salience network (SN) is associated with severity of symptoms and aberrant inter-network connectivity in major depressive disorder, (Menon, 2011; Manoliu et al., 2014; Wang et al., 2015). For instance, hyperactivity and hyperconnectivity of the DMN is frequently observed in depression (Hamilton et al., 2011, 2015) and thought to reflect disruption in passive self-referential processing (e.g., excessive rumination, negative attributions to self; Buckner et al., 2008). Atypical function and connectivity of the DMN has also been observed relative to individual differences in anxiety and in anxious psychopathologies (Simpson et al., 2001; Zhao et al., 2007; Gentili et al., 2009; Coutinho et al., 2015). Within the CEN, atypical function and communication may underlie deficits in cognitive functioning commonly observed in internalizing psychopathology. For example, impairments in executive control correspond to deficient recruitment of core regions of the CEN, such as the dlPFC, in individuals high in trait anxiety (Bishop, 2008; Pacheco-Unguetti et al., 2010; Basten et al., 2011), and decreased functional coupling within the CEN has also been demonstrated in patients with social anxiety disorder, compared to healthy controls (Liao et al., 2011; Qiu et al., 2011).

However, it is also important to consider how disrupted communication between the DMN and CEN may relate to psychopathology and personality. The antagonistic relationship between the DMN and CEN is facilitated by a separate network—the SN—which includes core regions such as the anterior insula, dorsal anterior cingulate cortex (dACC) and amygdala (Goulden et al., 2014; Menon, 2015). The SN has been proposed to detect the salience of incoming information to appropriately direct attention (Seeley et al., 2007; Menon, 2015); thus, the perceived salience of stimuli can have significant repercussions on attentional allocation and how internal and exogenous cues are processed. Moreover, functional connections between the SN and CEN have been demonstrated to underlie actual performance of cognitive tasks (Fang et al., 2016). Within the SN, the right anterior insula has been particularly noteworthy, as research has demonstrated that this region plays a causal role in initiating attentional switching between DMN and CEN states (Sridharan et al., 2008; Goulden et al., 2014). Given this role, inefficient communication between the anterior insula and nodes of either or both of these regions may result in difficulties shifting out of internally-focused processing and subsequently contribute to weaker performance on cognitively demanding tasks.

Indeed, the extant literature suggests that SN and anterior insula functioning is disrupted in internalizing psychopathology. Compared to healthy controls, individuals with depression have demonstrated decreased intra-network connectivity within the SN and decreased inter-network connectivity between the SN and DMN (Manoliu et al., 2014). For those with anxious pathology and related traits (e.g., neuroticism), hyperactivity of the insula appears to be a common feature (Wright et al., 2003; Mataix-Cols et al., 2004; Paulus and Stein, 2006). Increased connectivity within regions of the SN has also been found to relate to anxiety (Sylvester et al., 2012; Baur et al., 2013). Considering the role of the right anterior insula in attentional switching, disruption in SN may have profound downstream effects on appropriately integrating salient information to initiate CEN or DMN engagement.

Less is known about the specific relations between the SN and harm avoidance; however, several neuroimaging studies have revealed associations between harm avoidance and anterior insula functioning. Markett et al. (2013) demonstrated a positive association between harm avoidance and intra-SN connectivity between the anterior insula and dACC. In another study, Paulus et al. (2003) found that right anterior insula activation increased when participants made risky vs. safe decisions. Moreover, in this study, insular activation was modulated by subjects’ degree of harm avoidance, such that higher scores on this trait corresponded to greater magnitude of insula activation. Thus, although several neuroimaging studies have supported an association between the anterior insula and harm avoidance, to date, research has failed to investigate how complex internetwork communication may be modulated by individual differences in harm avoidance.

The goal of the current study was to examine the associations between harm avoidance and resting state functional connectivity. Given the role of the SN’s right anterior insula in modulating DMN vs. CEN states, the anterior insula was selected as a seed region to examine functional connectivity with both the DMN and CEN, as well as within the SN. Although the extant literature suggests that the right anterior insula, in particular, is critical in switching between the DMN and CEN (Sridharan et al., 2008; Goulden et al., 2014), several studies have indicated left hemispheric effects related to trait and state anxiety (Dennis et al., 2011; Baur et al., 2013). Accordingly, both left and right anterior insula were examined as seed regions to support continued examination of lateralized function and connectivity. The extant literature has suggested that harm avoidance is associated with increased intra-SN connectivity (Markett et al., 2013), while research within internalizing psychopathology relevant to harm avoidance has indicated excessive DMN (Hamilton et al., 2011, 2015) and deficient CEN connectivity (Bishop, 2008; Basten et al., 2011). This suggests that the anterior insula may not be appropriately modulating between attentional states, perhaps as a function of insufficient functional connections between the insula and these networks. As such, we hypothesized that harm avoidance would be: (1) positively correlated with connectivity between the anterior insula and other regions of the SN; and (2) negatively correlated with connectivity between the anterior insula and regions of the DMN and CEN. In order to examine specificity of these hypothesized effects, we utilized a self-report measure that more narrowly assesses harm avoidance as a behavioral inhibitory trait, and we also examined the association between trait anxiety and anterior insula-seeded resting state functional connectivity. Substantial research has also demonstrated aberrant activity and connectivity of the amygdala corresponds with anxiety (Rauch et al., 2003; Kim et al., 2011b; Baur et al., 2013). Thus to further explore differentiation between harm avoidance and trait anxiety, analyses were also conducted with an amygdala seed.

## Materials and Methods

### Participants and Procedure

Participants were 110 undergraduate students aged 18–35 (69 female) recruited from the University of Wisconsin-Milwaukee research subject pool. Eleven subjects were excluded for poor neuroimaging data quality (i.e., excessive motion during rest scan), resulting in a final analyzable *n* of 99. Participants were excluded from participation if they were left-handed, had any contraindications to magnetic resonance imaging (e.g., irremovable metal in body, pregnancy), or had a history of significant head trauma, neurological disorder, bipolar disorder, or psychotic disorder. Participants provided written informed consent after reviewing the study procedures. Study participation comprised completion of self-report questionnaires and magnetic resonance imaging (MRI) session. The scanning session lasted approximately 1.5 h and included structural and functional scans. Tasks completed in the scanner included fear conditioning/extinction, working memory and picture viewing tasks. Self-report measures were typically completed on a different day prior to the MRI scan, and functional resting state scans were collected at the end of the scanning session. All procedures were approved by the University of Wisconsin-Milwaukee and Medical College of Wisconsin Institutional Review Boards. All subjects gave written informed consent in accordance with the Declaration of Helsinki. Participants were compensated with course credit and $60 cash payment for their participation.

### Measures

#### Harm Avoidance

Harm avoidance was assessed using the harm avoidance subscale from the Multidimensional Personality Questionnaire (MPQ; Tellegen, 1985). The harm avoidance scale of the MPQ consists of 28 dichotomous self-report items. Nine items are true-false statements (e.g., “I would not like to try skydiving”). For the remaining 19 items, the respondent is asked to choose which of two situations they would like least (e.g., “Of the following two situations I would like least: (a) Walking a mile when it’s 15° below zero; (b) being near a volcano when it’s about to erupt”). The MPQ harm avoidance dimension has demonstrated good internal consistency (Patrick et al., 2002) and has also shown good specificity compared to other personality measures of harm avoidance, which may map onto traits such as negative emotionality or neuroticism rather than behavioral inhibition (Waller et al., 1991).

#### Trait Anxiety

The Trait version of the Spielberger State-Trait Anxiety Inventory (STAI; Spielberger et al., 1983) was used to measure trait anxiety. The STAI consists of 20 self-report items rated on a four-point scale. The STAI has demonstrated good psychometric properties, including high test-retest reliability and internal consistency (Barnes et al., 2002).

### MRI Data Acquisition

Imaging data was collected on a 3.0 Tesla short bore GE Signa Excite MRI system at the Medical College of Wisconsin. Functional T2*-weighted echoplanar images (EPIs) were collected for the resting state scan in a sagittal orientation: repetition time (TR)/echo time (TE) = 2,000/25 ms; FOV = 24 mm; matrix = 64 × 64; flip angle = 77°; slice thickness = 3.5 mm. Participants were instructed to remain still and to keep their eyes open while data was collected for 5 min.

For coregistration of the functional data, high resolution spoiled gradient recalled (SPGR) images were also acquired (TR/TE = 8,200/3.2 ms; FOV = 240 mm; matrix = 256 × 224; flip angle = 12°; voxel size = 0.9375 × 0.9375 × 1 mm).

### MRI Data Analysis

#### Image Processing

Resting state functional MRI (fMRI) data was analyzed using the CONN toolbox (Whitfield-Gabrieli and Nieto-Castanon, 2012). In preprocessing, EPI data was slice-time corrected to adjust for non-simultaneous slice acquisition within each volume. Images were corrected for head movements using a six-parameter (rigid body) linear transformation. Images were transformed to Montreal Neurological Institute space (MNI 152) and spatially smoothed to minimize effects of anatomical variability (FWHM = 6 mm). Linear detrending and temporal bandpass (0.01–0.1 Hz) filtering were performed to remove low-frequency drifts and high-frequency physiological noise (Cordes et al., 2001; Fox et al., 2005). Nuisance covariates including head motion parameters (and their first order derivatives), white matter signal and cerebrospinal fluid signal were regressed out (Cole et al., 2010).

Motion correction procedures in resting state functional connectivity analyses have become a prominent concern, as research has demonstrated that these analyses are particularly susceptible to spurious noise and distance-dependent changes in signal correlations caused by small head movements (Power et al., 2015). To reduce confounding effects of motion, frame-wise displacement (FD) was computed. Volumes with FD > 0.3 mm were “scrubbed” (i.e., excluded from further analysis), and participants with excessive motion (<4 min of useable data) were excluded from analyses.

To examine functional connectivity, both seed-to-voxel and independent component analyses (ICA) were conducted.

#### Seed-to-Voxel Functional Connectivity Analysis

For first-level seed-to-voxel analysis, the left (−44, 13, 1) and right anterior insula (47, 14, 10) from the CONN network atlas (Whitfield-Gabrieli and Nieto-Castanon, 2012) and left and right amygdala (anatomically derived from the automated anatomical labeling [AAL] toolbox; Tzourio-Mazoyer et al., 2002) were selected as seed regions. Mean BOLD time series were extracted from these seed regions and correlated with the time course of each voxel of the brain, resulting in a three-dimensional correlation coefficient (r) map for each subject and each seed. Normalized Fisher-transformed correlation maps were used for group analysis. Second-level seed-to-voxel analyses were conducted to allow for between-subjects comparisons. Subject connectivity maps were entered into a second-level general linear model to compare functional connectivity patterns as associated with: (1) MPQ-harm avoidance; and (2) STAI-trait anxiety scores. Sex and age were included as covariates in the model. The statistical threshold was set at *p* < 0.05 and corrected for multiple comparisons. The height threshold was set at *p* < 0.001 (uncorrected) and cluster-size threshold at *p* < 0.05 (FDR-corrected).

#### Seed-to-Network Functional Connectivity Analysis

An ICA was conducted to examine the spatial distribution of functional networks and their associated time courses. ICA analyses were employed in the CONN toolbox. Procedures included temporal concatenation across subjects and a principal component analysis (PCA) for group-level dimensionality reduction. The FastICA algorithm was used to extract 20 independent components. Identification of intrinsic functional networks was determined based on correlations between the spatial components and the CONN atlas, as well as visual examination of the spatial maps. Group-level maps were backprojected onto individual subject data to estimate subject-specific time courses and spatial maps. Sex differences in the spatial distribution of resting state networks were examined. To examine whether seed-to-network connectivity was modulated by individual differences in harm avoidance, the right and left anterior insula were selected as seeds in an ROI-to-ROI analysis where independent components for the DMN, SN and CEN served as other ROIs. Sex and age were included as covariates in the model. The statistical threshold was set at *p* < 0.05 (FDR-corrected).

## Results

### Participant Characteristics

Participant characteristics are provided in Table 1. There was a significant sex difference in harm avoidance, *t*_(97)_ = −4.74, *p* < 0.001, such that women reported higher levels of harm avoidance (*M* = 18.65, *SD* = 4.57) than men (*M* = 13.97, *SD* = 4.41). There were no significant differences in age or trait anxiety between males and females. Harm avoidance and trait anxiety were not significantly correlated in the sample (*r* = 0.15, *p* = 0.16).

**Table 1:** Sample characteristics (*n* = 99).

	Mean (SD) or %
Sex
Female	69.7%
Male	30.3%
Age	21.54 (3.34)
MPQ harm avoidance	17.23 (4.99)
STAI trait anxiety	40.31 (10.99)

*MPQ, Multidimensional Personality Questionnaire; STAI, Spielberger State-Trait Anxiety Inventory*.

### Functional Connectivity Results

#### Seed-to-Voxel Results

**Figure 1:**
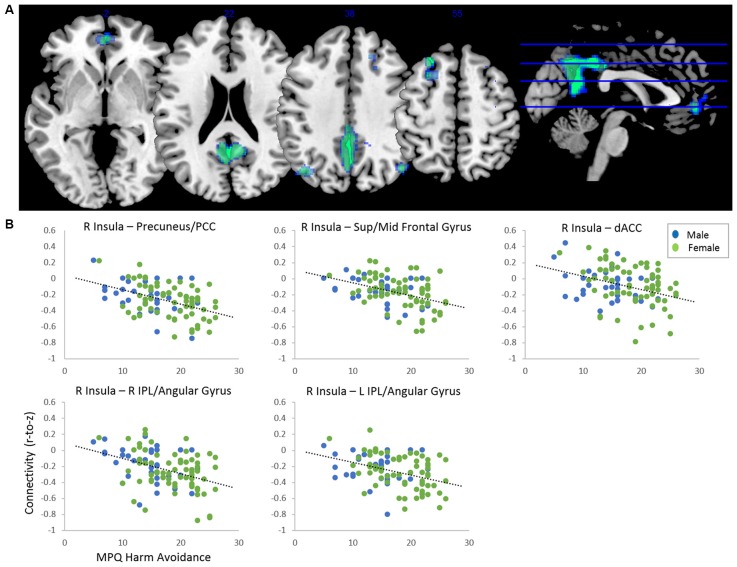
**(A)** Clusters showing significant negative effect of harm avoidance on connectivity to right anterior insula seed (*p* < 0.05 FDR-corrected, adjusted for sex and age). Clusters located within precuneus/posterior cingulate cortex (PCC; 6, −54, 22; *k* = 2,048), left superior/middle frontal gyrus (−24, 28, 54; *k* = 269), dorsal anterior cingulate cortex (dACC; 0, 48, −2; *k* = 181), right inferior parietal lobule (IPL; 46, −68, 38) and left IPL (−40, −70, 38; *k* = 238). **(B)** Scatterplots depicting functional connectivity (Fisher’s z) of the clusters plotted against harm avoidance scores from the Multidimensional Personality Questionnaire (MPQ).

After adjusting for sex and age, results indicated a main effect of MPQ harm avoidance on functional connectivity of both anterior insula seeds. These results are reported in Table 2 and Figure 1. For visualization purposes, connectivity (z) values from the significant clusters were extracted for each subject. For the right anterior insula, harm avoidance was negatively associated (i.e., stronger anticorrelations) with connectivity to clusters located in the precuneus/PCC (6, −54, 22; cluster size *k* = 2048), left superior and middle frontal gyrus (−24, 28, 54; cluster size *k* = 269), medial frontal gyrus/ACC (0, 48, −2; *k* = 181) and bilateral inferior parietal lobule (IPL)/angular gyrus (left: −40, −70, 38; *k* = 238; right: 46, −68, 38; *k* = 127). For the left anterior insula, harm avoidance was negatively associated with connectivity to a cluster in the precuneus/PCC (8, −32, 8; cluster size *k* = 432).

**Table 2:** Regions demonstrating decreased functional connectivity with increased harm avoidance.

Region	*k*	*t*_(95)_	*p*_FDR-corrected_	Peak coordinates (MNI)		
				*x*	*y*	*z*
**Right anterior insula seed**						
Precuneus/PCC	2,048	5.32	<0.001	6	−54	22
Superior/middle frontal gyrus (L)	269	4.50	0.001	−24	28	54
dACC	181	4.27	0.004	0	48	−2
IPL/angular gyrus (R)	127	4.18	0.012	46	−68	38
IPL/angular gyrus (L)	238	4.15	0.001	−40	−70	38
**Left anterior insula seed**						
Precuneus/PCC	858	4.80	<0.001	−6	−50	16

*PCC, posterior cingulate cortex; dACC, dorsal anterior cingulate cortex; IPL, inferior parietal lobule*.

There were no main effects of STAI trait anxiety on functional connectivity of the left or right anterior insula. There were no main effects of MPQ harm avoidance or STAI trait anxiety on functional connectivity of the amygdala.

#### Seed-to-Network Results

Results of the ICA revealed several networks of interest to the current analyses: anterior DMN (mPFC), posterior DMN (PCC, precuneus), left and right frontoparietal/CENs (frontal eye fields, dlPFC) and SN (insula, ACC). The effects of sex on the spatial distribution of functional networks was examined. There were no significant differences on any of the functional networks of interest between males and females.

In ROI-to-ROI analysis, harm avoidance was significantly negatively associated with connectivity between the right anterior insula and the components identified as the posterior, *b* = −0.02, *t*_(95)_ = −3.6, *p*-_FDR_ = 0.01, and anterior DMN, *b* = −0.02, *t*_(95)_ = −3.57, *p*-_FDR_ = 0.01. There were no effects of harm avoidance on connectivity between the insula and SN or CEN. There were also no effects of harm avoidance on connectivity between the amygdala and any of these networks of interest.

## Discussion

The current study examined the associations between harm avoidance and resting state functional connectivity of the anterior insula. Results indicated that harm avoidance was negatively associated with connectivity between the anterior insula and several clusters located within the DMN, CEN and SN. Effects of harm avoidance on connectivity were observed primarily based on the right anterior insula seed, consistent with research indicating the causal role of the anterior insula in modulating brain states is right lateralized (Sridharan et al., 2008; Goulden et al., 2014). However, a similar negative association between the left anterior insula and precuneus/PCC was also observed. Notably, no significant differences in connectivity were observed relative to individual differences in trait anxiety. Additional seed-to-network analyses indicated that there was a similar significant negative effect of harm avoidance on connectivity between the right anterior insula and components identified as the anterior and posterior DMN. Harm avoidance and trait anxiety were also not significantly correlated with each other. Thus, these findings support the notion that harm avoidance is a separable construct from general trait anxiety, and it appears that these processes are supported by different neurobiological substrates.

Moderating effects of harm avoidance were observed on internetwork connectivity between the SN’s anterior insula and DMN and CEN. Most notably, the observed negative associations between harm avoidance and connectivity reflected stronger anticorrelations between the anterior insula and DMN, including a large cluster within the precuneus/PCC. The precuneus/PCC has been established as a key node of the DMN (Fransson and Marrelec, 2008; Utevsky et al., 2014). Notably, while at rest, the precuneus/PCC has demonstrated higher metabolic activity than any other region of the brain (Gusnard and Raichle, 2001), suggesting that the precuneus/PCC is critical to the general internally-focused processes relevant to the DMN. Moreover, aberrant DMN activity and connectivity is common in internalizing psychopathology relevant to harm avoidance (Hamilton et al., 2011; Whitfield-Gabrieli and Ford, 2012). While anticorrelated activity between these regions is normal in healthy individuals (Fox et al., 2005; Uddin et al., 2008), it is possible that the increased magnitude of these anticorrelations reflects impairment in attentional allocation, such that excessive attentional resources are devoted to identified anxiety-provoking, behaviorally-relevant stimuli, making it difficult to disengage from these stimuli and return to baseline. Consistent with this idea, research has demonstrated that greater anticorrelations between the SN and DMN are associated with difficulties with emotion regulation (Rabany et al., 2017). As such, elevations in harm avoidance may be associated with deficits in attentional shifting, with the anterior insula being more easily triggered to processing external stimuli. Additional disruptions in internetwork connectivity likely further contribute to this deficit in attentional shifting for those high in harm avoidance, as connectivity was also reduced between the anterior insula and other regions such as the bilateral IPL and left middle and superior frontal gyrus.

Surprisingly, harm avoidance was associated with decreased connectivity within the SN, specifically between the anterior insula and dACC. Given the role of the SN in detecting the importance of incoming information in order to direct attentional resources (Menon, 2015), it had been hypothesized that perhaps increased attention to dangerous/threatening stimuli for those high in harm avoidance would have downstream consequences for how attentional resources are allocated. However, the current findings are consistent with some extant literature indicating that internalizing psychopathology, including depression and anxiety, is associated with decreased intra-SN connectivity (Liao et al., 2011; Manoliu et al., 2014). Notably, research on risk-taking has found that increased functional connectivity between the dACC and right anterior insula is associated with risky decision-making and externalizing behavior (e.g., alcohol/nicotine use; Claus et al., 2011, 2017; Wei et al., 2016). In light of the current findings, connectivity within this anterior insula-dACC circuit appears to index motivational/reward-seeking behavior, and thus the negative association between connectivity and harm avoidance may reflect a maladaptive propensity for risk aversion. However, other intra-SN circuits (e.g., insula-amygdala) have reliably demonstrated *increased* connectivity in those with internalizing psychopathology or high in trait anxiety (Rabinak et al., 2011; Baur et al., 2013). The current study did not find a positive association between anterior insula-amygdala connectivity and harm avoidance, suggesting that perhaps individual differences in harm avoidance do not affect the initial perceived salience of incoming information, but rather result from excessive attentional allocation to the anxiety-provoking stimulus and a struggle to disengage from it. As the current findings reflect functional connectivity while at rest inside the scanner, it is also possible that enhanced connectivity within certain circuits of the SN may only be present while in the actual presence of threat.

Contrary to *a priori* hypotheses, trait anxiety was not associated with any differences in connectivity of either the left or right anterior insula. One possible explanation for a lack of findings related to trait anxiety is that the sample comprised relatively healthy undergraduate students. Thus, anxious symptoms may not have been significantly elevated enough to correspond to differences in functional connectivity. It may be beneficial to examine whether trait anxiety relates to disrupted insular functional connectivity in clinical samples and whether these patterns differ from those related to harm avoidance. Alternatively, the current study may have failed to observe a main effect of trait anxiety on functional connectivity given the selection of the anterior insula as a seed region. In anxiety research, the extant literature has largely focused on the functional connections between the amygdala and prefrontal regions, such as the ventromedial PFC, proposed to downregulate hyperactive amygdala activity (Kim et al., 2011a). It is possible that trait anxiety is better characterized by disrupted communication between the amygdala and these inhibitory regions, rather than in dynamic interplay of DMN and CEN. It should be noted that in the current study, we also failed to observe modulation of amygdala-seeded functional connectivity by individual differences in both trait anxiety and harm avoidance. Again, this may be a limitation of the relatively healthy sample, and future work should aim to examine this question in more symptomatic samples. The STAI has also previously been criticized for its heterogeneity, as items of the STAI have been shown to map onto separate constructs related to anxiety/worry and sadness/self-deprecation (see Bieling et al., 1998). In light of the current findings, disrupted connectivity between the insula and DMN and CEN may be more relevant to the specific behavioral propensities captured by harm avoidance, while broader indices of trait anxiety may modulate different functional connections within the brain. Future work would likely benefit from further disentangling the complex features of internalizing psychopathology in order to better understand more precise neural mechanisms implicated in different facets of these phenotypes (e.g., cognition, behavior).

The current study has several limitations. First, the resting state fMRI scan was 5 min in duration. Emerging evidence has indicated that longer resting state scans produce more reliable data (Birn et al., 2013). Second, while the resting state design of the study helps provide initial evidence regarding the associations between harm avoidance and the interplay of intrinsic functional neural networks (as well as its discriminability from trait anxiety), future work would likely benefit from utilizing task-based designs to examine the functional connections between the anterior insula and DMN/CEN during tasks that may require attentional switching or simulate real-world behavioral inhibitory tendencies. Finally, the current study utilized a sample of relatively healthy college students. Although the sample included a good range of variability in regards to the harm avoidance and trait anxiety measures, it would likely be beneficial to have samples including those with clinical levels of internalizing symptoms in order to better characterize the potential mechanistic role of harm avoidance in these disorders. In addition, it would also be informative to examine the neurobiological correlates of harm avoidance for those *low* in harm avoidance, as this is also likely maladaptive and leads to reckless, harmful behavior. Indeed, harm avoidance has been found to be negatively correlated with components of psychopathy (e.g., antisocial behavior, callousness; Levenson et al., 1995; Gaughan et al., 2009).

Overall, the results suggest that increased harm avoidance is associated with disrupted functional connections between the anterior insula and regions of the DMN and CEN, suggesting individuals high in harm avoidance may experience difficulties in appropriately modulating attention between internally and externally focused processes. These findings were distinct from trait anxiety, for which there were no significant effects on anterior insula connectivity. As such, the behavioral inhibitory tendencies captured by harm avoidance may be uniquely relevant to individuals’ attentional switching abilities. Future work would likely benefit from continuing to disentangle the underlying neurobiological systems relevant to harm avoidance and its distinguishability from higher order personality traits (e.g., neuroticism) and anxiety.

## Data Availability

The raw data supporting the conclusions of this manuscript will be made available to any qualified researcher upon request.

## Author Contributions

AH performed data analyses and wrote the manuscript. EB, WP and TM acquired the data. All authors contributed to revision of the manuscript and interpretation of findings.

## Conflict of Interest Statement

The authors declare that the research was conducted in the absence of any commercial or financial relationships that could be construed as a potential conflict of interest.
